# Giant Intrahepatic Subcapsular Haematoma: A Rare Complication following Laparoscopic Cholecystectomy—A Case Report and Literature Review

**DOI:** 10.1155/2020/6410790

**Published:** 2020-10-19

**Authors:** Eltaib Saad, Lauren O'Connell, Anne M. Browne, W. Khan, R. Waldron, K. Barry, Iqbal Z. Khan

**Affiliations:** ^1^Department of General Surgery, Mayo University Hospital, Castlebar, Co. Mayo, Ireland; ^2^Department of Radiology, Mayo University Hospital, Castlebar, Co. Mayo, Ireland

## Abstract

We report on a 59-year-old female with symptomatic cholelithiasis on a background of morbid obesity who underwent an elective LC with an uncomplicated intraoperative course; however, she experienced a refractory hypotension within one hour postoperatively with an acute haemoglobin drop requiring fluid resuscitation and blood transfusion. A triphasic computed tomography scan revealed a large intrahepatic subcapsular haematoma (ISH) measuring 21 cm × 3.1 cm × 17 cm surrounding the lateral surface of the right hepatic lobe without active bleeding. She was managed conservatively with serial monitoring of haemoglobin and haematoma size. A follow-up ultrasound scan after eight weeks confirmed complete resolution of the haematoma. Giant ISH is a fairly rare, but life-threatening complication following LC which merits special attention. This case demonstrates the necessity of close postoperative monitoring of patients undergoing LC and considering the possibility of ISH, although being rare event, in those who experience a refractory postoperative hypotension. It also highlights the decisive role of diagnostic imaging in securing a timely and accurate diagnosis of post LC-ISH.

## 1. Introduction

Laparoscopic cholecystectomy (LC) is currently considered the gold standard for the management of symptomatic gallbladder disease [1, 2]. Advantages such as decreased hospital length of stay, reduced overall cost, improved postoperative pain, and increased patient satisfaction constitute the main reasons for the wide popularity of this minimally invasive approach [1, 2]. Specific complications still remain, in particular, bleeding, bile leak, and bile duct injury [1, 2]. However, intrahepatic subcapsular haematoma (ISH) represents a rare, but potentially life-threatening complication following LC [3–5]. Few cases have been reported in the literature so far [3–5]. The low incidence of this event would explain in part of the current limited experience for its diagnosis as well as the standard management [5]. Herein, we report a case of giant ISH following LC which was managed conservatively, and we reviewed all of previously published cases.

## 2. Case Presentation

A 59-year female presented to our outpatient clinic with recurrent episodes of right upper quadrant pain and nausea. Her medical history included morbid obesity (a body mass index (BMI) of 40.2), type II diabetes mellitus, hypertension, and right knee osteoarthritis. Her prescribed medications were metformin, atenolol, and buprenorphine patches. She was not on antiplatelets or anticoagulants. Previous surgical history included only an uneventful lumbar disc laminectomy. Ultrasound scan revealed fatty liver, multiple gallbladder stones without signs of cholecystitis, and a normal common bile duct (CBD) diameter. Preoperative work-up including full blood count, coagulation profile, and liver function tests was normal. An elective laparoscopic cholecystectomy was planned after discussing benefits and risks.

A prophylactic dose of enoxaparin (40 mg) was administered four hours preoperatively as per local protocol. An infraumbilical blunt port was inserted via Hasson' open technique. Following establishment of pneumoperitoneum, a further 10 mm epigastric operating port and two 5 mm right subcostal assisting ports were inserted under direct vision. The gallbladder was retracted, Calot's triangle anatomy was clearly displayed, and an adequate safety window was achieved before clipping and dividing the cystic artery and the cystic duct, respectively. The gallbladder dissection from the liver bed was accomplished without concerns by using an electric-coagulating hook. No blood loss or biliary leak was noted throughout the dissection. The gallbladder was extracted in an endobag through the infraumbilical port. All ports were removed under direct vision with no bleeding noticed from trocar sites. However, the patient had refractory hypotension within one hour postoperatively as her blood pressure dropped to 94/74 mmHg (compared to 140/90 mmHg one hour preoperatively) despite intravenous fluid resuscitation. She was saturating 97% on 100% oxygen therapy, with a pulse rate of 80 beats per minute and a respiratory rate of 20 breaths per minute. She was responsive and did not complain of abdominal pain. Abdominal examination revealed no signs of peritonitis. The haemoglobin level dropped from 13.5 g/dl preoperatively to 10.5 g/dl. Two units of red blood cells (RBCs) were transfused. Platelet count and coagulation profile were normal. An ECG showed T-wave inversion in leads II, III, and V1-V5; however, serial data (ECG and troponin levels) were negative for an acute coronary event. She was transferred to the intensive care unit for haemodynamic monitoring.

**Figure 1:**
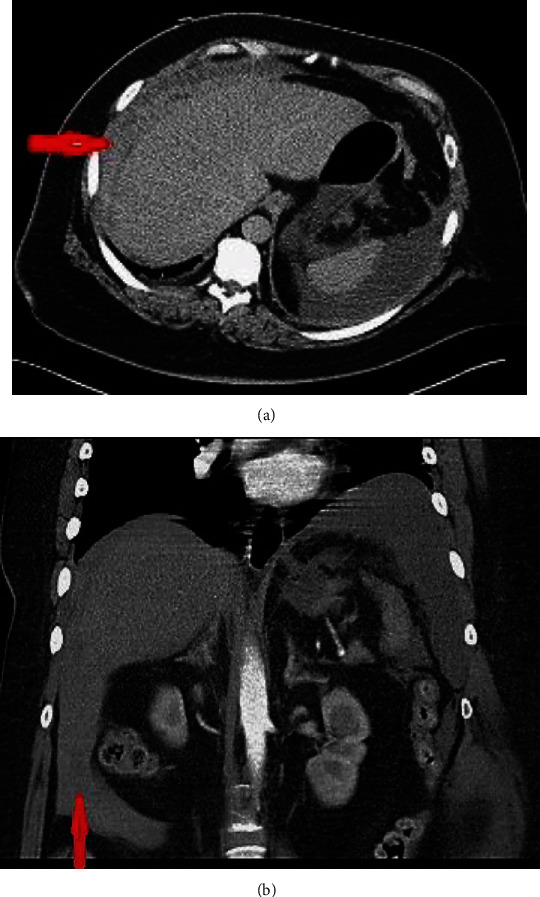
(a) Axial postcontrast CT image demonstrating acute subcapsular haematoma surrounding the lateral surface of the right lobe of liver (red arrow). (b) Coronal postcontrast CT image demonstrating ruptured subcapsular haematoma with fresh blood extending to the right paracolic gutter (vertical arrow points to a hyperdense material inferior to the liver).

Following haemodynamic stabilization, an urgent triphasic CT scan demonstrated a large (21 cm × 3.1 cm × 17 cm) acute subcapsular haematoma surrounding the lateral surface of the right lobe of the liver and extending to the anterior surface of the left lobe (Figures 1(a) and 1(b)), with fresh blood along the paracolic gutters bilaterally. No contrast extravasation was visualized, and the hepatic artery, portal vein, and hepatic veins opacified normally. She was transfused two more units of RBCs and required a pressor support to maintain haemodynamic stability overnight. Broad-spectrum intravenous antibiotics were commenced as per microbiology advice. A plan of laparoscopic exploration in the event of further haemodynamic instability was discussed with the patient and her family. A national hepatobiliary unit was consulted, and they advised to continue with the conservative management. The patient was transferred the following day to specialized hepatobiliary services for close observation and possible surgical or radiological intervention if deemed necessary.

She was continued on the conservative management in the tertiary care centre. The patient was discharged on day 7 upon stabilization of haemoglobin level and a partial resolution of the haematoma. A follow-up ultrasound scan eight weeks postoperatively demonstrated a complete resolution of the subcapsular haematoma.

## 3. Discussion

Despite LC being considered a largely safe operation with an overall morbidity of less than 7% [6], serious complications are well-documented with an incidence of 2.6% [2]. These include bleeding, bile duct injury, subhepatic abscess, and choledocholithiasis [1, 2, 6]. Postoperative bleeding is a fairly rare complication following LC occurring in less than 1% [5]. The most common sites of a possible bleeding following LC include the following: the gallbladder bed, the cystic artery, trocar insertion sites, the falciform ligament, and bleeding from liver capsule tears [5–8]. Occurrence of ISH following LC is a rare event, but it represents a life-threatening complication due to haemodynamic instability, and thus it merits special attention [5, 7].

According to Liu et al., only 16 cases of post-LC-ISH were reported in the literature between the years 1994 and 2005 [5]. Interestingly, all were females in keeping with this reported case. The time interval of diagnosis ranged widely from six hours up to six weeks [5]. Our patient experienced haemodynamic instability within one hour postoperatively which is presumably explained by the large haematoma size. Nearly half of the patients had haemodynamic instability on initial presentation, but all survived [5]. Various management modalities were followed depending on each patient's clinical status ranging from conservative management, radiologically guided percutaneous drainage, to laparoscopic exploration, and laparotomy which was indicated in seven patients [5].  Table 1 describes the characteristics of 18 cases of ISH following LC, as per our literature review up to the time of writing this report; it also summarizes the identified risk factors, the time interval to diagnosis, and the management given for each case. Owing to the rarity of ISH, no conclusive cause has yet been identified [5, 7]. A number of contributing factors have been postulated in the literature [3, 5, 7–12]. These include iatrogenic injuries to the liver parenchyma during excessive gallbladder manipulation or capsular tears during gallbladder traction [7, 8], anatomical variations of the hepatic vascular system such as pseudoaneurysm [9], and presence of subcapsular hepatic haemangioma that could be injured during gallbladder manipulation [7, 8]. Perioperative administration of nonsteroidal anti-inflammatory drugs (NSAID) was reported in a number of cases of ISH following LC with special emphasis on ketorolac use as it was associated with the highest risk estimate of bleeding [4, 10–12]. It was also reported in patients receiving anticoagulants [3].

In this reported patient, however, no specific underlying cause is evident for such a giant ISH, as she had no known bleeding or coagulation disorders and she was not taking anticoagulants or antiplatelet therapy. Furthermore, there was neither bleeding source identified nor liver parenchymal injury recognized throughout the operation. Nevertheless, the possibility of a subtle capsular tear cannot be definitively ruled out. It is possible that her BMI and presence of fatty liver at surgery may have also contributed. Additionally, there was no evidence of hepatic haemangiomas on the preoperative ultrasound scan. As a matter of fact, the exact cause of post-LC-ISH remained certainly unexplained in seven cases [5].

Notably, our patient had a remarkably giant ISH (21 cm × 3.1 cm × 17 cm) that was seldom described before. Furthermore, it was ruptured at the time of diagnosis similar to one case that was, however, associated with perioperative ketorolac administration and necessitated an exploratory laparotomy for haematoma evacuation due to persistent haemodynamic instability [12].

The management of ISH depends generally on the patient's clinical status and the haematoma size [5–7]. The reported patient became haemodynamically stable following fluid resuscitation, a pressor support, and blood transfusions. The risks of haematoma rupture should be carefully explained to the patients who are considered for a conservative approach, and the substantial need for a laparoscopic exploration and conversion to laparotomy have to be considered based on serial evaluations of a number of clinical, laboratory, and radiological parameters [5, 7], and of course, patient preference, surgeon experience, and facilities available locally. Furthermore, in patients who are haemodynamically stable, a radiologically guided percutaneous drainage of haematoma is a feasible choice if the haematoma size is too large and when systemic signs of haematoma infection exist [7, 12, 13]. One case described a selective embolization of an active bleeder in the right hepatic artery [3]. Nevertheless, the current experience relating to management of post-LC-ISH remains largely limited due to the rarity of the condition and paucity of the published cases.

## 4. Conclusion

Giant ISH is an extremely rare but life-threatening complication following LC. This case demonstrates the necessity of close monitoring of patients who undergo LC and considering the possibility of ISH, although being rare, in those who experience refractory postoperative hypotension.

## Figures and Tables

**Table 1:** Literature review of post-LC-ISH cases (1994-2019).

No	Authors/year of publication	Postulated risk/contributing factor	Time interval to diagnosis after LC	Management
1	Liu et al. 2019 [5]	No risk factor identified	Two days	Diagnostic laparoscopy and drainage tube placement
2	Moloney et al. 2017 [7]	Liver parenchymal injury	Two hours	Diagnostic laparoscopy prior to diagnostic imaging but the haematoma was managed conservatively
3	Głuszek et al. 2015 [14]	No risk factor identified	One day	Laparotomy and haematoma evacuation
4	Brown et al. 2015 [15]	No risk factor identified	Six days	Percutaneous US-guided drainage
5	de Castro et al. 2012 [3]	Anticoagulation use	Six weeks	Selective embolization followed by US-guided drainage of haematoma
6	Hansen et al. 2011 [13]	Liver capsule injury	Two days	Diagnostic laparoscopy and haematoma evacuation
7	Shibuya et al. 2010 [16]	Liver capsule injury and perioperative NSAID use	One day	Laparotomy and haematoma evacuation
8	Bravo et al. 2010 [4]	Perioperative ketorolac use/coagulation dysfunction associated with multiple myeloma	Six days	Laparotomy and haematoma evacuation
9	Bravo et al. 2010 [4]	Perioperative ketorolac use	One day	Laparotomy and haematoma evacuation
10	Shibuya et al. 2009 [17]	No definitive risk factor	Not available	Conservative management
11	Guercio et al. 2008 [12]	Perioperative ketorolac use	Not available	Laparotomy and haematoma evacuation
12	Shetty et al. 2005 [18]	Perioperative NSAID use	Three days	Percutaneous CT-guided drainage
13	Shetty et al. 2005 [18]	Perioperative NSAID use	Not mentioned	Conservative management
14	Bhandarkar et al. 2004 [19]	No risk factor identified	Ten days	Percutaneous US-guided drainage
15	Pietra et al. 1998 [10]	Perioperative NSAID use	One day	Percutaneous US-guided drainage
16	Pietra et al. 1998 [10]	Perioperative NSAID use	Seven hours	Laparotomy and haematoma evacuation
17	Obara et al. 1998 [20]	No risk factor identified	Not mentioned	Conservative management
18	Erstad and Rappaport 1994 [11]	Perioperative NSAID use	Nine hours	Laparotomy
